# Community Case Study on Trauma-Specific Treatment and Counseling for Refugee Women Exposed to Intimate Partner Violence

**DOI:** 10.3389/fpsyt.2019.00891

**Published:** 2019-12-05

**Authors:** Anneke Pogarell, Susan Garthus-Niegel, Amera Mojahed, Clara von Verschuer, Ute Rokyta, Wenke Kummer, Julia Schellong

**Affiliations:** ^1^Psychotherapy and Psychosomatic Medicine, University Hospital Carl Gustav Carus, TU Dresden, Dresden, Germany; ^2^Department of Child Health, Norwegian Institute of Public Health, Oslo, Norway

**Keywords:** mental health, refugee women, intimate partner violence, posttraumatic stress disorder, trauma-specific psychotherapy, aid network, counseling, post migration

## Abstract

Women experiencing intimate partner violence (IPV) are at high risk to suffer from severe mental health consequences, such as posttraumatic stress disorder (PTSD) and depression. Refugee women being exposed to IPV in the country of arrival are an especially vulnerable and understudied group and post migration persistent IPV should not be underestimated. Hence, research on special requirements regarding the treatment of these women is needed. We describe two individual cases from our work with refugee women suffering from PTSD symptoms who experienced IPV representing our trauma-specific therapeutic approach targeting this population. By analyzing their personal and medical history as well as their interactions with several institutions of the public sector and counseling centers, we illustrate the possibilities and limitations when helping our clients dealing with trauma-related mental health problems following the experience of IPV. Furthermore, we formulate general recommendations for providing adequate therapeutic frameworks concerning special requirements for the work with refugee women.

## Introduction

Intimate partner violence (IPV) is defined as the experience of psychological, physical, and/or sexual abuse from current or previous male or female partners (1). Exposure to IPV is associated with an increased risk of developing mental health problems, including posttraumatic stress disorder (PTSD) and depression (2–4).

According to the World Health Organization (WHO) globally one in three women has experienced either physical and/or sexual violence, in most cases by an intimate partner. Available research indicates that immigrant and refugee women are especially vulnerable to IPV (5, 6), as they are often exposed to established risk factors of IPV, such as originating from a community with broken social and protective networks, low levels of women’s access to paid employment, and dependencies on their intimate partners due to legal regulations (6, 7).

However, trauma-specific treatment according to official guidelines and counseling for refugee women as victims of IPV involves special requirements, as this population might have particular problems, such as difficult housing circumstances and language barriers (8–10). These special requirements need to be pointed out and discussed in order to overcome social inequalities and barriers to health care for refugee women being exposed to IPV. Implementation of requirements is needed to improve the mental health of refugee women in general (11).

## Background and Rationale

Our outpatient clinic for psychological trauma at the Dresden University Hospital Carl Gustav Carus is specialized in the treatment of PTSD and other trauma-related mental disorders. Most of our patients receive treatment paid for by their statutory health insurance and represent a population suffering from multiple mental health issues including dissociative or complex PTSD elements. If patients reach out to our facility shortly after being exposed to IPV or violent crimes in general, their treatment can also be paid for by public means for crime victims’ compensation. As IPV is considered to be a violent crime in Germany, victims are entitled to compensation under the Crime Victims’ Compensation Act (12). This includes up to 15 probationary sessions with a psychotherapist to provide fast access to the help system. If needed, further psychotherapeutic treatment can be provided by the services paid by the statutory health insurance.

**Figure 1:**
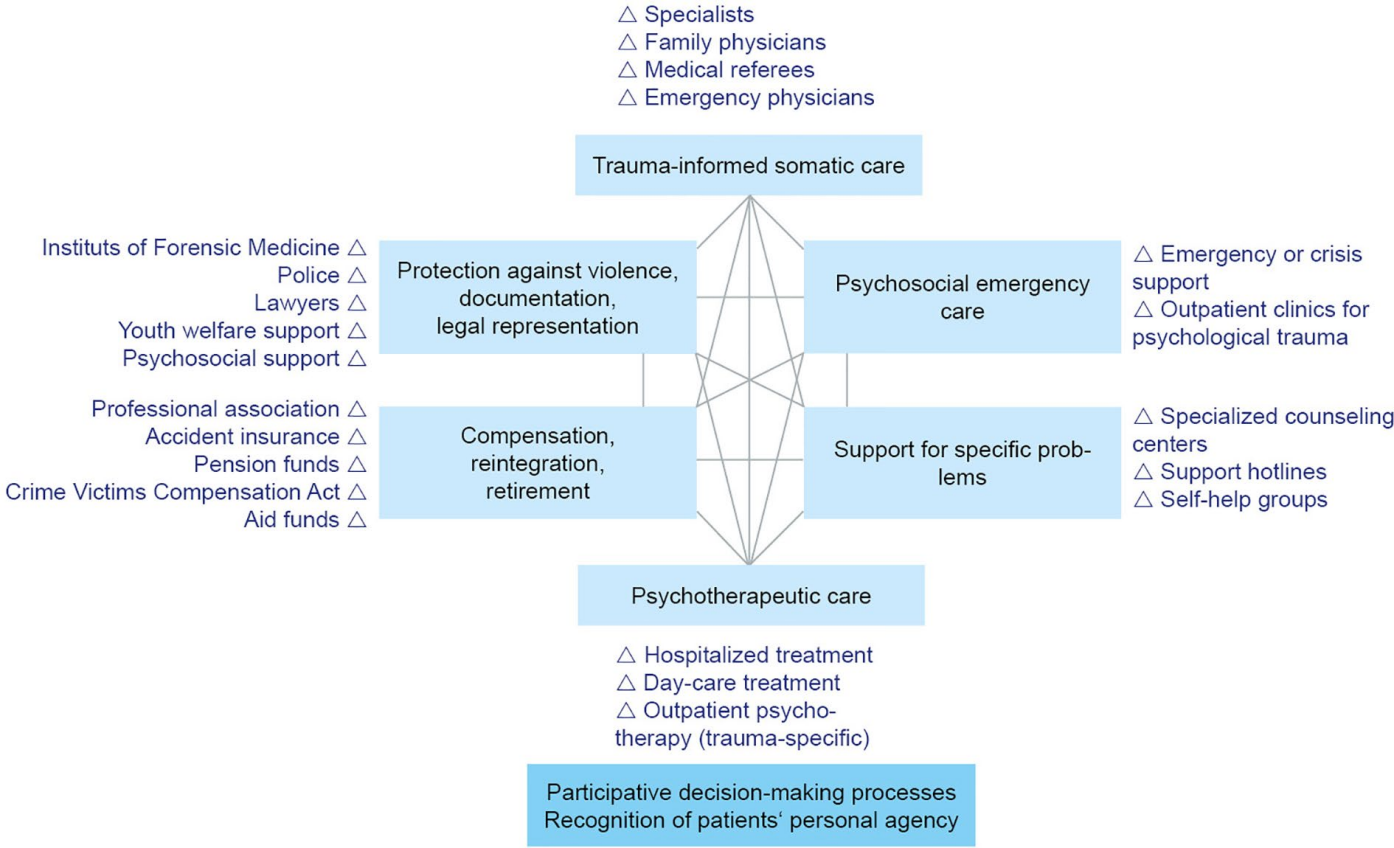
Topic areas and operators in the networking care for traumatized patients adapted from Reference (13).

Within the diverse population of our clinic’s patients, refugee women are a special group with specific needs. In recent years, the number of refugee women among our clients has been increasing, presenting us with the challenge to implement the common therapeutic approach in working with this population and meeting their special needs. **Figure 1** provides an overview of our general therapeutic approach.

## Methodological Aspects

### Data Base

The data used for this article are derived from our internal clinic information system, where the clinic staff (psychologists, nurses, physicians, social workers) document every interaction with the patient as well as their observations. The authors contributed to collecting the data over the course of the treatment of the subjects in our facility. We obtained informed written consent from the patients authorizing the publication of their clinical cases.

### Subjects and Methods

For this case report, we presented two complexly traumatized patients from our outpatient clinic whose mental health problems are typical for refugee women having been exposed to IPV in Germany (the country of arrival). Admission, diagnostic, and treatment processes were described, social and legal implications were listed for each case, results of each intervention that led to their current health improvement were described, challenges over the course of their treatments were also mentioned. By analyzing these factors of the therapeutic process, we showed what is needed to apply our general therapeutic approach for complexly traumatized patients to the special population of refugee women suffering from IPV in the country of arrival.

We aim to further the knowledge about the process of treatment and counseling of refugee women with PTSD and exposure to IPV. We want to present a strategy for other medical facilities offering treatment for the population of the study, hoping to contribute to overcome the barriers to access to health care services for these women.

**Figure 2:**
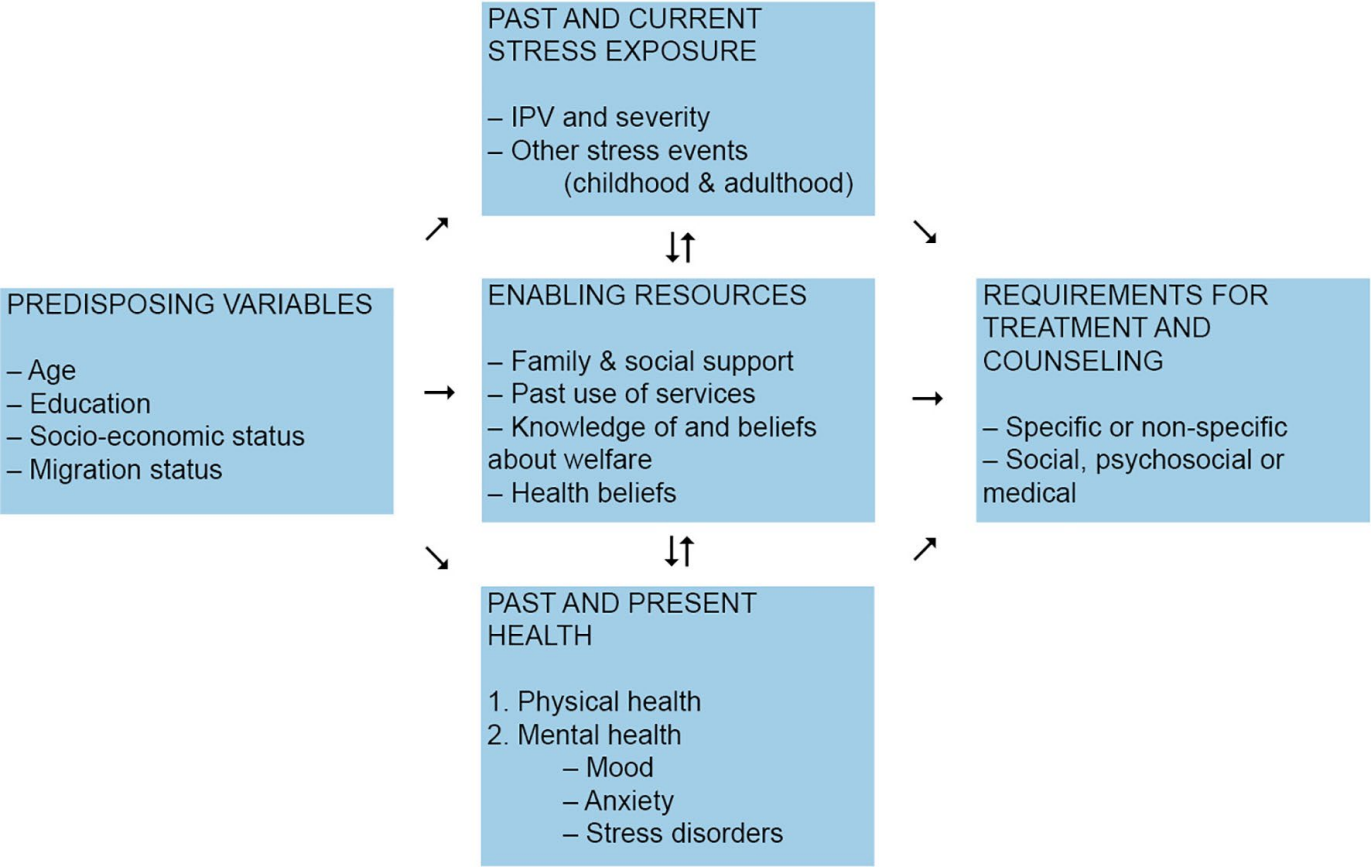
Requirements for treatment and counseling for victims of IPV adapted from Reference (14).

In this article, we present two case reports. Both of the presented clients are diagnosed with PTSD following the exposure to severe IPV. They represent the challenges and enabling factors regarding treatment and counseling in this specific population. These challenges can be illustrated as depicted in **Figure 2**.

#### Subject I

**Figure 3:**
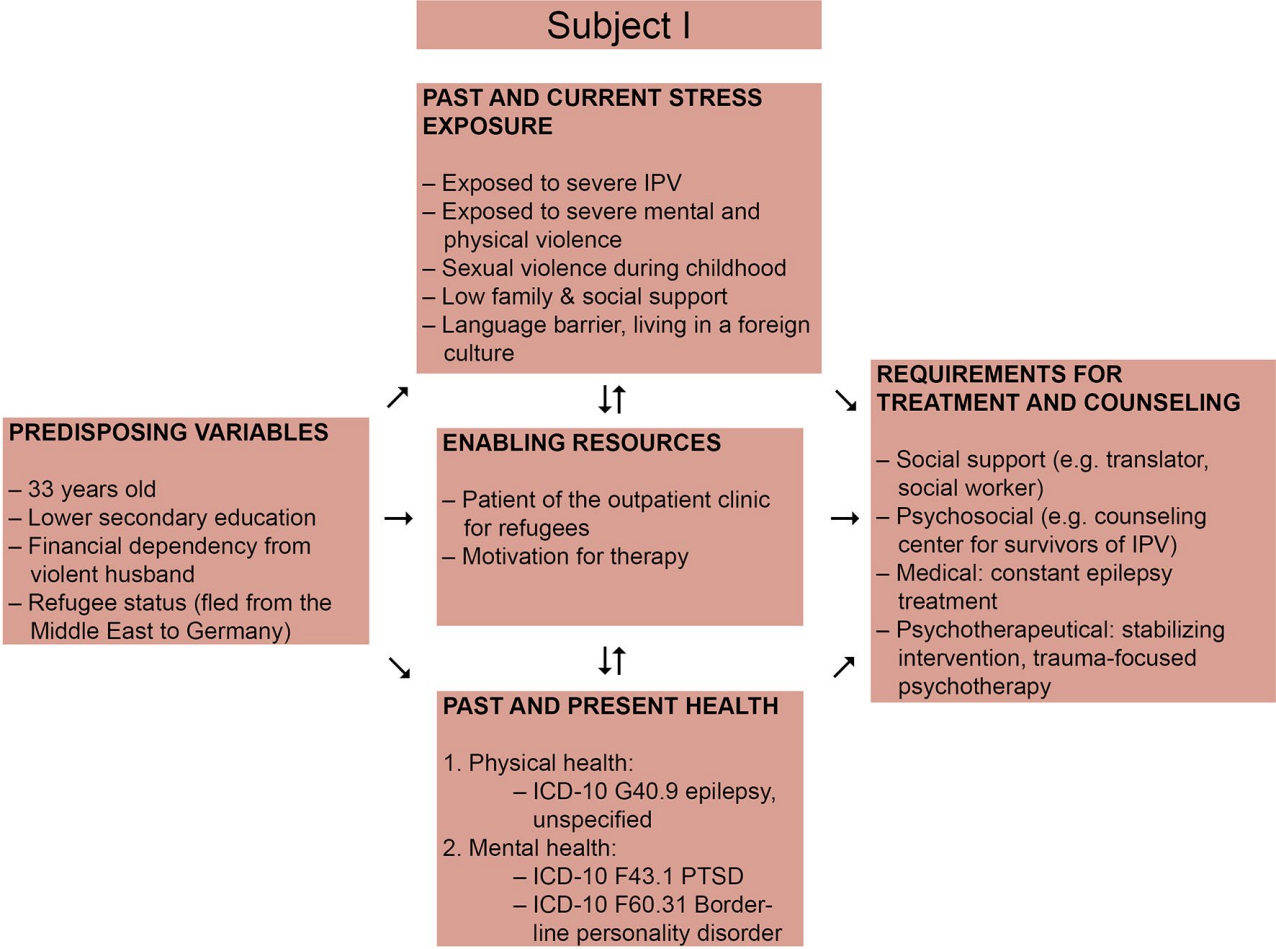
Requirements for treatment and counseling for victims of IPV adapted from Liang et al., 2005, presenting the case of Subject I (14).

The first patient is a woman from a country in the Middle East, who was 33 years old at the time of admission. We illustrate an overview of the challenges and enabling factors regarding treatment of this patient in **Figure 3**. She was referred to our outpatient clinic for Psychotraumatology at the Dresden University Hospital by an outpatient clinic for refugees because of attempted suicide, intrusions, and nightmares. Besides, she had been suffering from epilepsy since her childhood. The patient reported to have been growing up in a violent family, being exposed to sexual and physical violence by her uncle and brother. When she could no longer bear the violence, she fled from the house and decided to escape her country of origin. On her way to Germany she fell into the hands of smugglers and decided to get married to protect herself from assaults by other men. The present relationship with her husband was violent as well. She reported not to be able to attend German language courses as she was anxious and therefore avoided leaving her apartment.

Although suffering from mental health problems since her childhood, the patient never received psychiatric or psychotherapeutic treatment before. For epilepsy treatment she had been prescribed antiepileptics (Lamotrigin 200 mg 1–0–1 and Levetiracetam 500 mg 1–0–1).

In the mental health status examination, it was observed that the patient appeared suspicious und distanced. Intrusions existed as thoughts and flashbacks. She appeared to be emotionally unstable showing impulsive affect regulation. She seemed to be highly tensed and in an unhappy-dejected mood. At the time of the examination the patient sufficiently distanced herself from suicide. There was no indication of abusive consumption of addictive substances or addiction.

All criteria for Posttraumatic stress disorder (PTSD, ICD-10 F43.1) were met at this first clinical interview. Moreover, based on reported psychosocial and biographical details, Borderline personality disorder (ICD-10 F60.31) was suspected additionally to the previously diagnosed epilepsy (ICD-10 G40.9).

It took two years to establish a stable therapeutic relationship enabling the use of trauma-specific techniques according to guidelines. Crisis intervention and contact to specific counseling centers for IPV were at the center of the first therapy sessions because of the persistent IPV perpetrated by the husband. Effective cooperation with a local specialized counseling center for IPV was of invaluable help. We held several case conferences with their consultants to coordinate psychosocial help and to avoid double structures. As we were always dependent on the aid of an interpreter at our appointments, additional expenditure of time for organizational matters regarding the interpreter service was needed.

Over the course of the treatment, several therapy breaks occurred. Incidents like further IPV exercised by the husband or attempted suicide led to an admission at an inpatient department at a different hospital. During this period, antidepressant (Agomelatine) and mood-stabilizing neuroleptic (Quetiapine) medication was initiated. It often happened that the patient did not attend scheduled appointments without an excuse or that she was late. Hence, we set up a contract of punctuality, which led to higher reliability. During the course of the treatment at our clinic, the patient regularly consulted our clinic’s physician. Medication was adapted several times, as the former antidepressant medication had led to adverse side effects. Trimipramine was tolerated best.

During the individual psychotherapeutic therapy, we kept on exploring biographical details and helped the patient deal with current symptoms. We also focused on reflecting on her personality as we analyzed everyday life conflicts together. It continued to be difficult to focus on trauma therapy due to many organizational matters. The patient herself experienced frustration and anger, and often showed a demanding attitude towards other people.

For a longer period of time, the patient consulted our clinic’s social worker frequently as she had troubles finding a new apartment to finally be able to physically separate from her abusive husband, whom she described as controlling and aggressive. She and her violent partner were registered as a ‘community of dependence’, implying that they had to support each other financially. Furthermore, the patient sought help as her social benefits were cut, because she had not attended any integration course.

Two years after she first came to our facility she finally became financially independent from her partner. The groundwork has now been laid to apply trauma-specific treatment techniques in order to improve the patient’s quality of life.

#### Subject II

**Figure 4:**
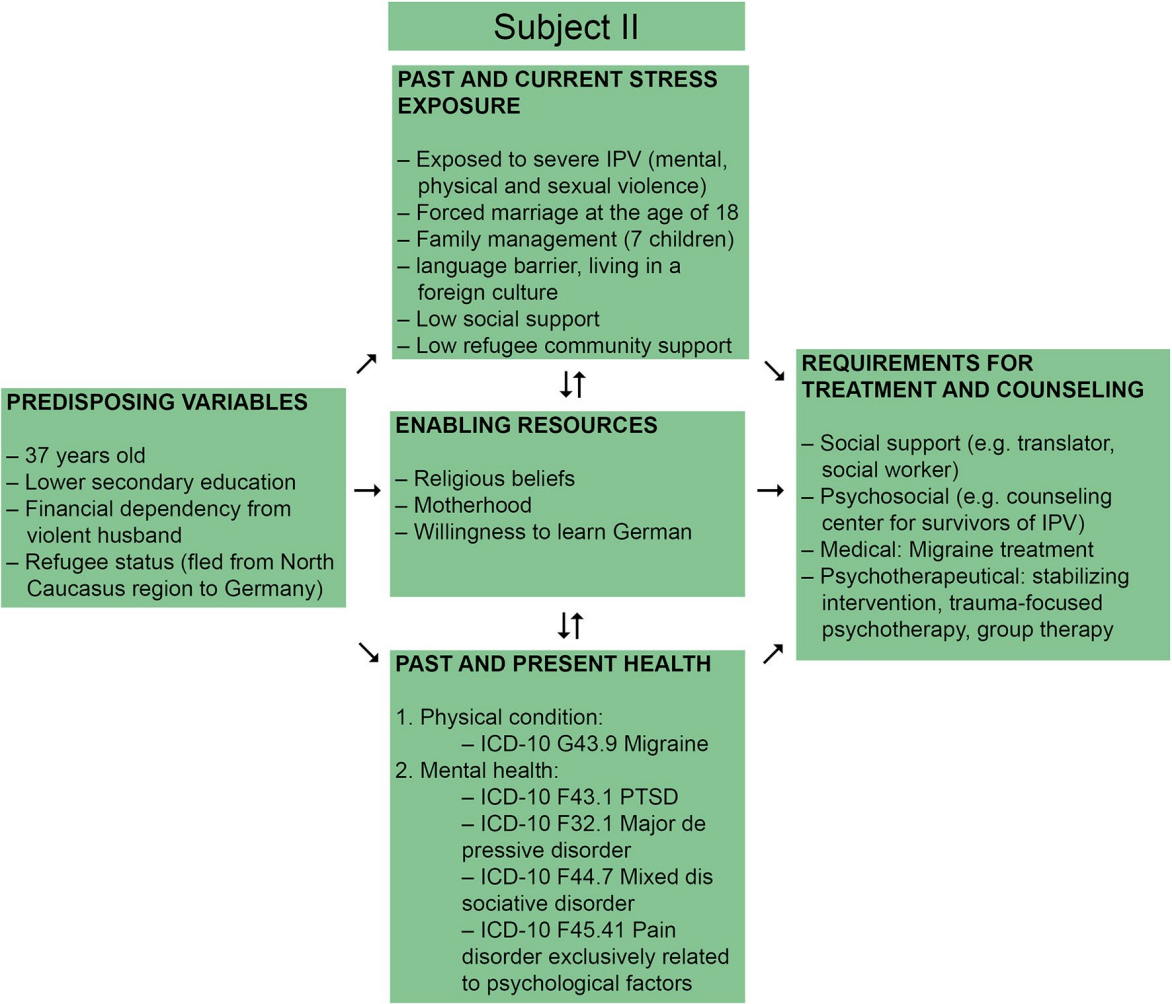
Requirements for treatment and counseling preferences for victims of IPV adapted from Liang et al., 2005, presenting the case of Subject II (14).

Our second patient is a refugee woman from the North Caucasus region, who was 37 years old at the time of admission. We illustrate an overview of the challenges and enabling factors regarding treatment of this patient in **Figure 4**. She reported having mental health problems following forced marriage at the age of 18 and abusive family relationships, but she never received treatment before. She had no previous history of medical conditions. The patient was referred to the outpatient clinic for psychological trauma at the Dresden University Hospital Carl Gustav Carus for the first time by her general practitioner (GP). At that time, she was living together with her violent and abusive husband and her six children. She was unintentionally pregnant and living in constant fear of her extremely violent husband, who was beating both her (also during her pregnancies) as well as their children.

She reported a long history of IPV including mental and physical violence as well as severe sexual violence since the beginning of the forced marriage to her husband. During her first pregnancy, she was beaten up by her husband so badly that she had to go to the hospital. However, her husband did not let her get medical treatment. During the time when she was pregnant with her sixth child her husband forced her to hand-dig a well in their garden at minus 10°C. She would always have to work, even at night, to earn money for the family. She told us that her partner would use that money to buy alcohol and drugs so that she and her children only had very little to eat. The patient tried to get separated from her husband in her country of origin. But because her children were then taken away from her and sent to members of her husband’s family she eventually came back to him. Because her husband got in trouble in their country of origin, he decided to take the family to Germany. She had to follow him, in the beginning with the hope that he would treat her better in Germany and stop consuming drugs. But his behavior did not change and she became pregnant again in Germany. In the fourth month of her last pregnancy her husband beat her up so badly that a social worker from another organization noticed it and helped reporting him to the police and referred her to a GP.

In the mental health status examination, the patient seemed to be very shy and tense. She reported symptoms of insomnia and chronic headaches as well as amnestic dissociative episodes. Intrusions existed as thoughts and flashbacks of IPV experiences; when she woke up after a nightmare she felt the pain physically. Her mood was depressed, she showed low motivation on the one hand, and seemed to be highly tensed regarding psychomotoric functioning on the other. She reported sometimes having suicidal thoughts but being able to distance herself from those thoughts sufficiently. There was no indication of abusive consumption of addictive substances or addiction.

As a result of the mental health status examination, we diagnosed her with severe Major Depressive Disorder (ICD-10 F32.1) and PTSD (ICD-10 F43.1) with dissociative and complex symptomatology; both diagnoses were not related to the pregnancy (Z34.8). Mixed dissociative disorder (ICD-10 F44.7) and pain disorder exclusively related to psychological factors (ICD-10 F45.41) were diagnosed additionally as these symptoms were persistent two years after birth of the seventh child.

As she was pregnant, when she first came to our clinic, we decided to offer her crisis intervention, including psychoeducation and low-dose antidepressants in order to improve sleep quality (Amitriptylin 25 mg 0–0–0–1/2). Intense social care in parallel with specific IPV counseling helped her separate from her partner, with the arrangement of the divorce, finding a new apartment, and other organizational matters. The youth welfare center was involved as the children had to be protected. A restraining order against the husband was imposed. The parental rights of the ex-partner were terminated, only accompanied contact between father and children was allowed. Still he threatened her several times, saying that he would kill her or take away her children.

After giving birth to her seventh child, trauma-specific psychotherapy was paused for almost two years. As PTSD and dissociative symptoms persisted for those two years, trauma-specific psychotherapy was continued. Besides enhancing her understanding of anamnestic and biographical details as well as working with defining the trauma focus, resource oriented techniques for dealing with the dissociative and somatic symptoms were installed. Sometimes longer therapy breaks occurred as one of the patient’s children became severely sick. The patient reported feeling socially ostracized as the community of people from her country of origin would judge her for leaving her husband and not wearing a hijab anymore. The paternal side of her family would be judging her for ‘leaving her family’ and said that she was the ‘black sheep of the family that has to go away’. Therefore, the patient reported to be very scared of going back to her country of origin.

During the group therapy the patient had the opportunity to learn to use relaxation techniques as well as to benefit from the exchange with others and learn from their experiences. The patient reported this to be very helpful and was open to join other participants of the group therapy for social events outside the therapy setting. Still, she remained in great need of further guideline based trauma-specific treatment.

## Results

We presented the cases of two refugee women suffering from PTSD after being exposed to IPV. Both of the cases were managed with pharmacotherapy, psychotherapeutic sessions (in one case also group therapy interventions), and counseling with social workers. Both patients showed typical patterns of refugee women with PTSD caused by IPV experiences.

As the analysis of the treatment and counseling requirements showed, the predisposing variables for both subjects are adverse. On admission, both patients suffered from persistent stress exposure, limited enabling resources, and adverse physical and mental health conditions. Both patients were multi-morbid regarding their mental health condition. It is for these reasons, that both subjects needed a lot of support not only from Physicians and psychotherapists but also from social workers and interpreters. Before we could begin with a trauma-specific psychotherapy we provided stabilizing psychotherapy and put a lot of effort to build a stable relationship with the patients, which led to an additional expenditure of time compared to therapeutic processes with other populations.

## Discussion

As we pointed out in our results section, there are special requirements for the treatment of refugee women with complex history of trauma including exposure to IPV. In the following, we want to discuss two of the aspects that we find most important regarding an adequate psychotherapeutic and counseling approach.

### Need for Perseverance

As our case reports show, the treatment and counseling of women suffering from mental health problems following IPV require a remarkably large amount of additional expenditure of time, which is confirmed by other studies regarding refugee mental health (15). Persistent violence is an enormous obstacle to the application of trauma-focused therapy. In the presented cases several years of resource oriented therapy and support by social workers were needed as trauma-specific treatment approaches were not feasible at that time (8). It is important to point out that this may cause frustration in the clinicians, and in the women, too; as it might appear that there is no progress at all, e.g., the process of terminating the relationship with a violent partner is often complicated and dangerous (16, 17). It is especially difficult for refugee women, due to financial and legal limitations. Parental responsibility for joint children is also a factor that increases the likelihood of continuing victimization, even if restraining orders are imposed (17). It is very important for clinicians to be aware of the women’s emotional distress relating to termination of a violent relationship. Furthermore, affected women often report experiencing a lack of support from their families and their social environment (18), which is confirmed by our examples. As seen in Subject I, women who grew up in a violent home are more likely to become victims of IPV, too (19).

In the presented cases, both women suffered from mental health problems after having been exposed to severe IPV. However, IPV is not the only form of violence most of these patients have experienced. Women fleeing from their country of origin are very likely to experience physical and/or sexual assault during their flight (20, 21). This can also be seen in the case of Subject I, who married somebody just to protect herself from assaults from other men. Such exposure to violence makes the diagnostic process more complex and requires more demanding therapy. A complex trauma history can also lead to attachment disorders, the patients then have difficulties in building stable relationships, also with the clinicians.

During the often prolonged course of therapy clinicians need the opportunity to reflect on the therapeutic process, e.g., in regular case conferences, and remind each other that it is normal that treatment in these cases may take a long time. Recent research and our experience shows that a combination of a variety of therapeutic interventions and counseling is needed to meet the requirements of these complex cases (22).

### Extensive Aid Network

As there are usually several institutions involved in helping the victims of IPV, it is important to be informed about each other’s interventions (23). With the consent of our patients we regularly hold case conferences with counseling centers, external social workers etc.

We found that the ongoing support from social services is extremely important for refugee women experiencing or having experienced IPV. First, there are many challenging organizational matters that not only the women are unable to solve alone (due to their mental health problems or language barriers), but that also represent barriers for starting trauma-specific treatment. In both of the presented cases the women needed a lot of support from the social workers to get separated from their violent partners, get legal arrangements, and to become financially independent as well as to get the opportunity to live separately from their abusive partner. Moreover, additional support by social services, as well as stable work and living conditions are highly correlated with decreased reports of chronic pain (24).

When working with mentally ill refugee women, aid from an interpreter is usually needed (25). In Germany, however, interpreter-aided therapy is not available for everyone. If the asylum proceedings are still pending or if the women are recognized refugees, interpreter-aided therapy can usually proceed. If their status is unclear, e.g., they are granted subsidiary protection, interpreters can only be paid privately. In conclusion, there is a group of refugee women, who do not have access to interpreter-aided therapy. In addition, it is time consuming for the clinic’s staff to organize interpreters for every appointment, even if the service is covered financially. The therapeutic success of the therapy also depends on the relationship not only between therapist and patient, but also between patient and interpreter.

Since we only presented two cases of a wider population and against the background of a lack of studies on this vulnerable group, caution is needed when generalizing our results. Researchers’ own subjective feelings may influence the reporting of the case study (researcher bias). We found that our patients suffered from multi-morbidities, however such multi-morbidities make causal claims difficult, i.e., claiming PTSD symptoms to be a direct consequence of IPV (26, 27). However, it is important to mention that dealing with multi-morbidities and PTSD reflects the everyday reality of outpatient clinics for psychological trauma.

The studies that do exist in connection with psychotherapeutic support for refugees show the importance of appropriate housing and access to specialized help and counseling networks with low barriers to entry, as well as broader social integration. Caretakers for refugees should be flexible and well-trained in trauma-informed care, but also attentive to the need for stable material living conditions, as mediated by social services (11, 28, 29). All of this equates to a significantly higher investment of resources, for psychotherapy to be applied with any measure of success.

Through our own professional experience, we have come to an understanding that even more resources than usually available are required to provide extensive support of social workers and interpreters in facilities giving treatment to refugee women with PTSD and exposure to IPV. Abusive relationships are often persistent even over the course of therapy and the patients need a lot of support for leaving the partner perpetrating IPV. The lack of these services might be a barrier to access to health services those women need urgently and to provide PTSD treatment according to guidelines. Furthermore, future studies of this particular population are necessary to gain deeper knowledge of the special requirements for the therapy of PTSD in these women in order to improve their quality of life.

## Conclusion

As has been shown in both individual cases, patients of this population are often characterized by multi-morbidities and require long-term multi-professional care.

We demonstrated that the general psychotherapeutic approach for the treatment of PTSD can be applied to refugee women with PTSD and exposure to IPV. However, it became clear that the therapeutic approach needs to be adjusted to the special needs of this population.

In the future, further studies are needed in order to better explore and understand the dynamics of IPV in this population and to tackle/reduce its specific consequences on refugee women.

## Data Availability Statement

The datasets generated for this study are available on request to the corresponding author.

## Ethics Statement

Ethical review and approval was not required for the study on human participants in accordance with the local legislation and institutional requirements. The patients/participants provided their written informed consent to participate in this study. Written informed consent was obtained from the individual(s) for the publication of any potentially identifiable images or data included in this article.

## Author Contributions

AP, SG-N and JS contributed to the conception as well as the design of this article and did the literature search. AM gave methodological advice. CV, UR, and WK were involved with counseling and treating the subjects of the case reports. CV contacted and asked them to allow us to use their stories. UR and WK reported their therapeutic experience with the subjects. AP extracted and synthesized the data from the medical reports and wrote the first draft of the manuscript. JS contributed her expertise as a physician specialized in trauma related mental health problems. All authors contributed to manuscript revision, read, and approved the submitted version.

## Funding

The primary author is related to the project ‘Kompetenzzentrum Traumaambulanzen in Sachsen’ (center of excellence for the treatment of psychological trauma in outpatient clinics in Saxony). Grant by the federal state of Saxony.

We acknowledge support by the open access publication funds of the SLUN/TU Dresden.

## Conflict of Interest

The authors declare that the research was conducted in the absence of any commercial or financial relationships that could be construed as a potential conflict of interest.
